# Epigenetic reprogramming, gene expression and *in vitro* development of porcine SCNT embryos are significantly improved by a histone deacetylase inhibitor—*m*-carboxycinnamic acid bishydroxamide (CBHA)

**DOI:** 10.1007/s13238-014-0034-3

**Published:** 2014-03-14

**Authors:** Yuran Song, Tang Hai, Ying Wang, Runfa Guo, Wei Li, Liu Wang, Qi Zhou

**Affiliations:** 1 State Key Laboratory of Reproductive Biology, Institute of Zoology, Chinese Academy of Sciences, Beijing, 100101 China; 2 Graduate University of the Chinese Academy of Sciences, Beijing, 100049 China

**Keywords:** swine, nuclear transfer, epigenetic reprogramming, histone deacetylase inhibitor

## Abstract

Insufficient epigenetic reprogramming of donor nuclei is believed to be one of the most important causes of low development efficiency of mammalian somatic cell nuclear transfer (SCNT). Previous studies have shown that both the *in vitro* and *in vivo* development of mouse SCNT embryos could be increased significantly by treatment with various histone deacetylase inhibitors (HDACi), including *Trichostatin A*, *Scriptaid*, and *m*-carboxycinnamic acid bishydroxamide (CBHA), in which only the effect of CBHA has not yet been tested in other species. In this paper we examine the effect of CBHA treatment on the development of porcine SCNT embryos. We have discovered the optimum dosage and time for CBHA treatment: incubating SCNT embryos with 2 μmol/L CBHA for 24 h after activation could increase the blastocyst rate from 12.7% to 26.5%. Immunofluorescence results showed that the level of acetylation at histone 3 lysine 9 (AcH3K9), acetylation at histone 3 lysine 18 (AcH3K18), and acetylation at histone 4 lysine 16 (AcH4K16) was raised after CBHA treatment. Meanwhile, CBHA treatment improved the expression of development relating genes such as *pou5f1*, *cdx2*, and the imprinted genes like *igf2*. Despite these promising *in vitro* results and histone reprogramming, the full term development was not significantly increased after treatment. In conclusion, CBHA improves the *in vitro* development of pig SCNT embryos, increases the global histone acetylation and corrects the expression of some developmentally important genes at early stages. As in mouse SCNT, we have shown that nuclear epigenetic reprogramming in pig early SCNT embryos can be modified by CBHA treatment.

**Electronic supplementary material:**

The online version of this article (doi:10.1007/s13238-014-0034-3) contains supplementary material, which is available to authorized users.

## Introduction

Somatic cell nuclear transfer (SCNT) has been a successful technology to derive cloned animals in many species (Wilmut et al., 1997; Kato et al., 1998; Wakayama et al., 1998; Baguisi et al., 1999; Onishi et al., 2000; Chesne et al., 2002; Shin et al., 2002; Galli et al., 2003; Woods et al., 2003; Zhou et al., 2003; Lee et al., 2005; Li et al, 2006). It is a valuable tool in fundamental research, biomedical and reproduction fields (Verma et al., 2011; Bendixen et al., 2013). As pigs share much similar physiological characteristics with human beings, it is a potential animal model for human disease and xenotransplantation (Gock et al., 2011; Ekser et al., 2012; Luo et al., 2012). SCNT is a practical way to produce targeted genetic modification in pigs.

However, like in other animals, SCNT in pigs is inefficient, due to the low cloning efficiency, fetus abnormality, and placenta deficiency (Polejaeva et al., 2000; Walker et al., 2002). It is widely believed that the crucial cause of poor developmental capacity in nuclear transfer embryos is the aberrant nuclear reprogramming. There is a close relationship between embryo reprogramming and epigenetic modification. DNA methylation and histone acetylation are the main modification patterns in epigenetic reprogramming. Compared with normal fertilized zygotes, in SCNT embryos nuclear, reprogramming has to occur within a short time span, so a more “open” chromatin configuration may be beneficial to SCNT embryos (William et al., 2001; Whitworth et al., 2010; Ogura et al., 2013). Histone deacetylase inhibitor (HDACi), has been proved to increase the acetylation level in somatic cells or in nuclear transfer embryos, as it makes the chromatin more flexible and allowing the combination of transcriptional factors (Kishigami et al., 2006; Lager et al., 2008; Dai et al., 2010).

In pigs, some histone deacetylase inhibitors, such as Trichostatin (TSA), *Scriptaid*, and Valproic acid (VPA), have been proved to increase the *in vitro* development of SCNT embryos (Li et al., 2008; Miyoshi et al., 2010; Zhao et al., 2010; Kim et al., 2011). Our previous study has shown that *m*-carboxycinnamic acid bishydroxamide (CBHA), a member of type II HDACi, could improve the *in vitro* and full term development of mouse SCNT embryos and improve the cell line establishment efficiency of embryonic stem cells (ES) derived from SCNT embryos (SCNT-ESCs) (Dai et al., 2010). However, CBHA has not yet been studied in domestic animals. In this study we use this HDACi to treat porcine nuclear transfer embryos to investigate whether it affects the developmental efficiency in pigs.

We have examined the optimum treatment program of CBHA on pig nuclear transfer embryos and observed the development rates in various treated and non-treated groups. To further explore how it improves the development of porcine nuclear transfer embryos, we detected the global acetylation levels of Histone 3 at Lysine 9 (AcH3K9), Histone 3 at Lysine 18 (AcH3K18), and Histone 4 at Lysine 16 (AcH4K16) using immunofluorescence in the CBHA treated embryos and control embryos. Then we analyzed the effect of CBHA treatment on the transcription level of some development-related genes, histone deacetylase genes, and imprinted genes using quantitative PCR. Finally, we detected the full term developmental efficiency of CBHA treated and non-treated embryos.

## Results

### *In vitro* development of porcine SCNT embryos are improved by CBHA treatment

To test whether CBHA could improve the early development of porcine SCNT embryos, we treated the SCNT embryos with different concentrations of CBHA and calculated the developmental rates from 2-cell to blastocyst stage (Table 1). We found that proper CBHA treatment had no effect on the cleavage rates of porcine SCNT embryos, but could significantly increase the *in vitro* development rate to blastocyst stage. The optimum treatment concentration of CBHA was 2 μmol/L, while 200 μmol/L or higher concentrations were detrimental to embryos. After treatment with 2 μmol/L CBHA for 24 h, the blastocyst ratio of CBHA-SCNT embryos was two folds (26.5%) of the embryos in control group (12.7%).

**Table 1:** Pre-implantation development of pig SCNT embryos under treatment with different CBHA concentration

CBHA Treatment (μmol/h)	No. of reconstructed embryos	No. of 2-cell (% reconstructed)	No. of blastocysts (% 2-cell)	No. of blastocysts (% reconstructed)
0	212	132 (62.3)	27 (20.5)	27 (12.7 ± 5.7)^a^
0.02/24	58	40 (69.0)	8 (20.0)	8 (13.8 ± 1.4)^a^
0.2/24	123	75 (61.0)	25 (33.3)	25 (20.3 ± 9.0)^b^
2/24	211	117 (55.4)	56 (47.9)	56 (26.5 ± 12.3)^c^
10/24	90	60 (66.7)	17 (28.3)	17 (18.9 ± 17.9)^b^
20/24	213	110 (51.6)	35 (31.8)	35 (16.4 ± 10.7)^b^
50/24	90	35 (38.9)	12 (34.3)	12 (13.3 ± 3.8)^a^
200/24	65	1 (1.5)	0 (–)	0 (–)
400/24	65	0 (–)	0 (–)	0 (–)

Significant **χ**^2^ comparisons: a versus c, *P* < 0.05; a versus b, *P* > 0.05; b versus c, *P* > 0.05

Next, to optimize the protocol for CBHA treatment, we examined the effect of different CBHA incubation times on the blastocyst ratios of SCNT embryos. The SCNT embryos were treated with 2 μmol/L or 10 μmol/L CBHA for 16 h, 20 h, and 24 h, respectively. There was no significant difference among the three groups, but the treatment of 2 μmol/L, 24 h gave the best blastocyst ratio (Table 2).

**Table 2:** Effect of time duration of CBHA treatment on the pre-implantation development of SCNT embryos

Concentration of CBHA (μmol /L)	Time duration (h)	No. of reconstructed embryos	No. of 2-cell (% reconstructed)	No. of blastocysts (% 2-cell)	No. of blastocysts (% reconstructed)
2	16	77	40 (52.0)	11 (27.5)	11 (14.3 ± 12.5)
	20	78	44 (56.4)	17 (38.6)	17 (21.8 ± 3.8)
	24	77	34 (44.2)	16 (47.1)	16 (20.8 ± 7.2)
20	16	73	34 (46.6)	13 (38.2)	13 (17.8 ± 13.2)
	20	80	38 (47.5)	13 (34.2)	13 (16.3 ± 13.9)
	24	78	36 (45.2)	10 (27.8)	10 (12.8 ± 4.0)
0	–	80	33 (41.3)	14 (42.4)	14 (17.5 ± 6.7)

### Blastocyst quality was improved after CBHA treatment

**Figure 1:**
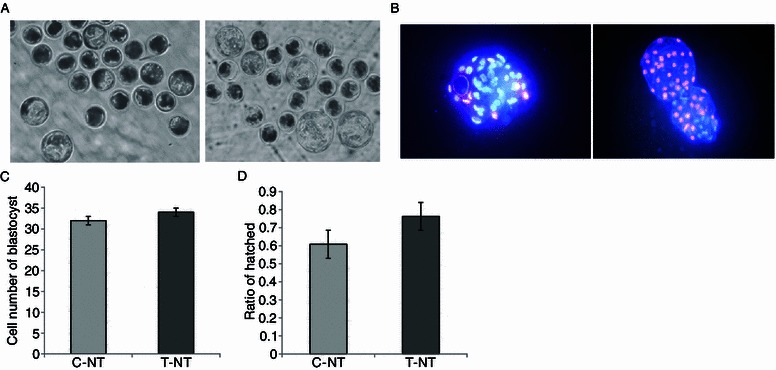
**Effect of CBHA on hatching rate and cell number of SCNT blastocysts**. (A) Representative photographs of pig blastocysts. Day 7 blastocysts developed from 0 μmol/L CBHA treated SCNT embryos (C-NT group) and 2 μmol/L CBHA treated SCNTembryos (T-NT group). Original magnification was 100×. (B) Fluorescent photographs of pig blastocysts stained with Hoechst. Original magnification was 200×. (C) Statistics of the number of cells in blastocyst from 0 μmol/L CBHA treated SCNT embryos (C-NT group) and 2 μmol/L treated embryos (T-NT group). (D) Ratio of hatched blastocysts from 0 μmol/L CBHA treated SCNT embryos (C-NT group) and 2 μmol/L treated embryos (T-NT group)

To unravel the improvement effects of CBHA treatment on SCNT embryo development, we examined the blastocyst cell number in two groups (Fig. 1). Though the cell numbers in the treated group were higher than the non-treated group, there was no significant difference between the two groups (34 vs. 32). Cell numbers from both groups were lower than their *in vivo* and *in vitro* fertilized counterparts.

Embryo implantation and further development are largely related to the blastocyst-hatching rate and the communication between embryo and the uterus. Hence we examined the blastocyst-hatching rate in the two groups. The hatched blastocysts were observed under a stereomicroscope at 168 h after activation (Fig. 1). Although the hatching rate in the CBHA treated group (76.25%) was much higher than those in the control group (67%), there was no significant difference between these two groups. Nevertheless, we found that embryos treated with CBHA could reach the blastocyst stage much earlier than those in the control groups, indicating that at the developmental phase they are more like the *in vivo* embryos, who reach the blastocyst stage on about day 5.

### CBHA treatment increases global histone acetylation levels of pig SCNT embryos

**Figure 2:**
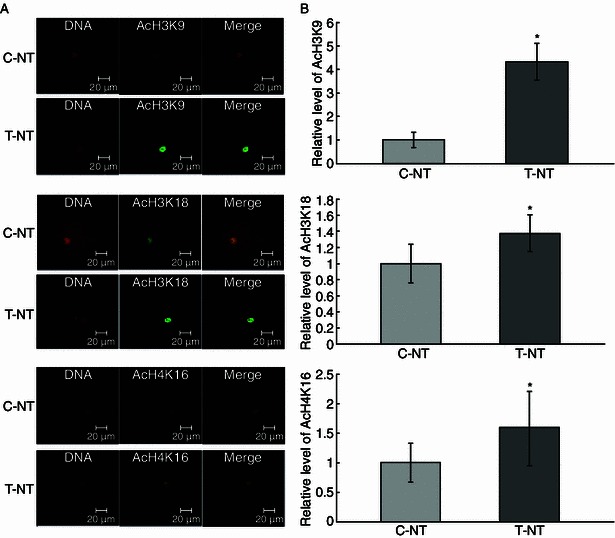
**The global AcH3K9, AcH3K18, and AcH4K16 levels of 1-cell stage embryos**. (A) Staining of AcH3K9, AcH3K18, and AcH4K16 (green) in non-treated control SCNT embryos (C-NT), and 2 μmol/L CBHA treated embryos (T-NT) at 1-cell stage. Each sample was counterstained with DAPI to visualize DNA (red). Original magnification was 200×. (B) Quantification of AcH3K9 and AcH3K18 signal intensities in C-NT (gray bars) and T-NT (black bars) embryos. Labeling intensity was expressed relative to that of the C-NT embryos (set as 100%). *P < 0.05. The experiments were replicated 3 times. *n* = 8–10 per group

**Figure 3:**
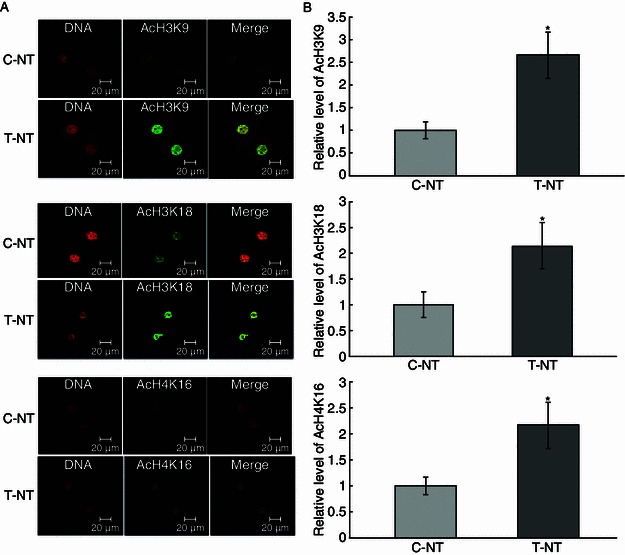
**The global AcH3K9, AcH3K18, and AcH4K16 levels of 2-cell stage embryos**. (A) Staining of AcH3K9, AcH3K18, and AcH4K16 (green) in non-treated control SCNT embryos (C-NT), and 2 μmol/L CBHA treated embryos (T-NT) at 2-cell stage. Each sample was counterstained with DAPI to visualize DNA (red). Original magnification was 200×. (B) Quantification of AcH3K9 and AcH3K18 signal intensities in C-NT (gray bars) and T-NT (black bars) embryos. Labeling intensity was expressed relative to that of the C-NT embryos (set as 100%). *P < 0.05, ^†^*P* < 0.01. The experiments were replicated 3 times. *n* = 8–10 per group

**Figure 4:**
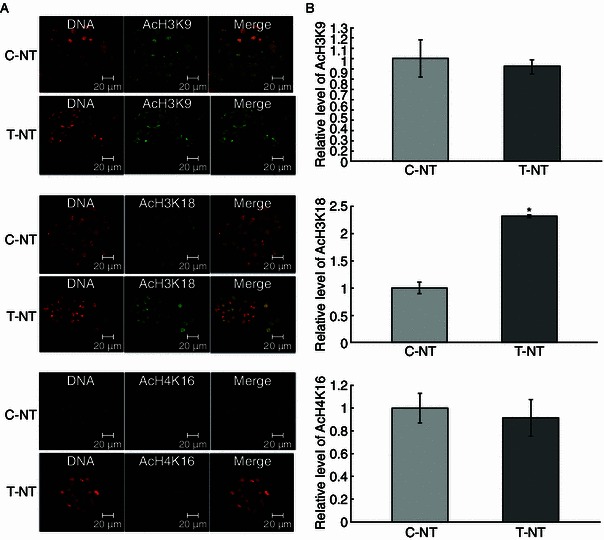
**The global AcH3K9, AcH3K18, and AcH4K16 levels of blastocyst stage embryos**. (A) Staining of AcH3K9, AcH3K18, and AcH4K16 (green) in non-treated control SCNT embryos (C-NT), and 2 μmol/L CBHA treated embryos (T-NT) at blastocyst stage. Each sample was counterstained with DAPI to visualize DNA (red). Original magnification was 200×. (B) Quantification of AcH3K9 and AcH3K18 signal intensities in C-NT (gray bars) and T-NT (black bars) embryos. Labeling intensity was expressed relative to that of the C-NT embryos (set as 100%). *P < 0.05. The experiments were replicated 3 times. *n* = 8–10 per group

As shown in embryos, the global histone acetylation level is closely related to the reprogramming state of chromatin. Meanwhile, we showed that an open, decondensed chromatin state was relevant to the pluripotency of iPSCs (induced pluripotent stem cells) and ESCs (embryonic stem cells). We further examined the embryo histone acetylation levels by assaying three epigenetic markers: AcH3K9, AcH3K18, and AcH4K16 at 1-cell, 2-cell, and blastocyst stages. We found that CBHA treatment increased the level of AcH3K9, AcH3K18, and AcH4K16 at 1-cell (Fig. 2) and 2-cell stages (Fig. 3). However, apart from AcH3K18, at the blastocyst stage there were no differences between the control and experiment groups (Fig. 4).

### Relative transcription level of development related genes, imprinted genes, and histone deacetylase genes

**Figure 5:**
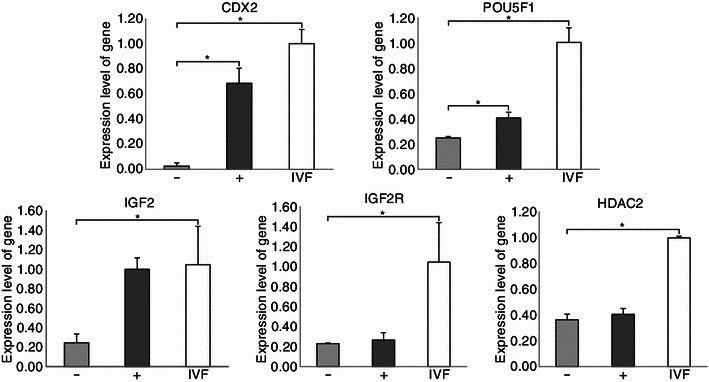
**Relative expression at the transcriptional level of histone deacetylase genes, development-related, and imprinted genes**. Blastocysts, IVF (open bars), non-treated control SCNT embryos (C-NT) (gray bars), and 2 μmol/L CBHA treated embryos (T-NT) (black bars) were collected on day 7. *P < 0.05; *n* = 15

In mammalian early embryonic development, *pou5f1* (*oct4*), an indicator for inner cell mass, is widely accepted to be a key factor in embryonic quality and pluripotency maintenance. Meanwhile, *cdx2*, a pivotal trophectoderm (TE) marker, is important for placenta formation. The expression level of imprinted genes and histone deacetylase genes is also vital for nuclear reprogramming. So, the transcription levels of the five genes (*pou5f1*, *cdx2*, *igf2*, *igf2r*, and *hdac2)* whose functions are mentioned above were detected by quantitive PCR (Fig. 5) at the blastocyst stage. The expression of two key developmental genes, *pou5f1* and *cdx2,* was significantly increased by CBHA treatment. However, the expression of these two genes in both groups was significantly lower than the IVF (*in vitro*, fertilized) group. The expression of the imprinted gene *IGF2* was increased after the CBHA treatment, which was similar to the IVF ones and significantly higher than the control group. However, the expression of another imprinted gene, *igf2r*, and the histone deacetylase gene, *hdac2*, was not affected by CBHA treatment, and both of them were significantly lower than the IVF ones.

### Full term development of porcine SCNT embryos following CBHA treatment

To determine whether CBHA could improve the full term development of porcine SCNT embryos, 2-cell stage embryos with and without CBHA treatment were transferred to the oviducts of surrogates on the day of or 1 day after the onset of estrus. In the first batch of experiments, we transferred more than 200 embryos to each surrogate. We found that there was no significant difference between the two groups (Table 3), which is inconsistent with the beneficial effect of CBHA in the *in vitro* development. To further exclude the noise caused by the excessive number of embryos transferred, we designed a second batch of experiments, which transferred only 100 embryos to each recipient (Table 4). We obtained a total of 15 live, 6 dead, and 1 mummy from the CBHA treated group and 17 live, and 7 dead from the control group. The full term development ratio of CBHA-treated embryos was 4.2%, which is not significantly different to the control group (4.4%), suggesting CBHA treatment does not improve the full term development of porcine SCNT embryos.

**Table 3:** Full-term development of CBHA treated SCNT embryos

Cell type	No. of transferred embryos	CBHA treatment^a^	Pregnancy check^b^	Piglets born	Cloning efficiency (%)^c^
PEFCs	231	+	−	0	0.94
PEFCs	187	+	+	2 dead + 1 mummy	
PEFCs	276	+	+	1 live + 1 dead + 2 mummy	
PEFCs	263	+	+	1 live + 1 dead	
PEFCs	262	−	−	0	
PEFCs	280	−	+	2 live + 5 dead	1.12
PEFCs	259	−	+	1 live + 3 dead	
PEFCs	269	−	+	2 live + 1 dead	
PEFCs	272	−	+	1 dead	

^a^+, Treated; −, untreated

^b^+, pregnant; −, not pregnant

^c^No. of piglets/No. of embryos transferred

**Table 4:** Full-term development of CBHA treated SCNT embryos with minimum transfer volume

Cell type	No. of transferred embryos	CBHA treatment^a^	Pregnancy check^b^	Piglets born	Cloning efficiency (%)^c^
PEFCs	100	+	+	6 live + 3 dead + 1 mummy	4.2
PEFCs	100	+	+	6 live + 3 dead	
PEFCs	100	+	+	3 live	
PEFCs	100	+	−	0	
PEFCs	100	+	−	0	
PEFCs	100	−	+	5 live + 1 dead	4.4
PEFCs	100	−	+	5 live + 1 dead	
PEFCs	100	−	+	2 live	
PEFCs	100	−	+	5 live + 3 dead	
PEFCs	100	−	−	0	

^a^+, Treated; −, untreated

^b^+, pregnant; −, not pregnant

^c^No. of piglets/No. of embryos transferred

## Discussion

Nowadays, somatic cells can be reprogrammed in many ways, the most common being somatic cell nuclear transfer (SCNT) and induced pluripotent stem cells (iPSCs) (Wilmut et al., 1997; Kato et al., 1998; Wakayama et al., 1998; Baguisi et al., 1999; Onishi et al., 2000; Chesne et al., 2002; Shin et al., 2002; Han et al., 2003; Galli et al., 2003; Woods et al., 2003; Zhou et al., 2003; Lee et al., 2005; Li et al, 2006; Takahashi et al., 2006; Yu et al., 2007). After being transfected with pluripotent genes or small molecules, a fraction of somatic cells could be reprogrammed to a pluripotent state like embryonic stem cells (ESCs). However, it has been reported that there are differences in the epigenetic modification and expression of genes between iPSCs and ESCs and the expression of exogenous pluripotent factors is likely to cause tumors (Okita et al., 2007; Bock et al., 2011; Menendez et al., 2011). Although several iPS cell lines have been reported, the silencing of exogenous genes and germline transmission remain unsolved. Thus nuclear transfer is still an indispensable method for somatic cell reprogramming. Meanwhile for pigs, besides therapeutic cloning, SCNT is a useful tool in transgenic research and especially in xenotransplantation and disease models. However, the efficiency of SCNT is still low and the mechanism of nuclear reprogramming is poorly understood.

To improve the efficiency of reprogramming, numerous factors or molecules have been used to alter the epigenetic modification in somatic cells or reconstructed embryos. HDACi is a widely used epigenetic modifier in cancer treatment and somatic cell nuclear reprogramming such as SCNT and iPSCs derivation. However, not all HDACi are beneficial to SCNT embryos, with some of them being unprofitable or detrimental. Generally, type II HDACi is used in epigenetic reprogramming (Monteriro et al., 2011).

Here we used CBHA, a hybrid polar compound, to improve the development of pig SCNT embryos. We found that the efficiency of SCNT and the quality of SCNT embryos are improved after CBHA treatment. Compared with non-treated embryos, CBHA treated SCNT embryos showed a higher global histone acetylation level. Expression of the development-related genes and the imprinted genes at blastocyst stage was also improved with the CBHA treatment.

In mouse SCNT, the optimum CBHA treatment protocol is 20 μmol/L incubation for 10 h post activation (Dai et al., 2010). However, in pig SCNT, we found that the best protocol was 2–10 μmol/L treated for 20–24 h after activation. This difference may be due to the differing developmental time spans between mice and pigs.

Histone acetylation is a significant epigenetic modification in embryo development and nuclear reprogramming. In previous studies, it has been shown that global hyperacetylation associated with a more permissive chromatin state which is an indicator of more fully reprogramming state in nuclear transfer and iPSCs (Li et al., 2002; Rybouchkin et al., 2006). In iPS cells, compared to partially reprogrammed ones, the fully reprogrammed iPSCs exhibit a more open and decondensed chromatin state and are more identical to ES cells (Mattout et al., 2011). We tested the histone acetylation level of reconstructed embryos after the CBHA treatment to examine the effect of this HDACi. Compared with the control SCNT embryos, the CBHA SCNT embryos showed a higher level of global histone acetylation. AcH3K9, AcH3K18, and AcH4K16 were all increased at the 1-cell and 2-cell stages, but there were no significant differences at the blastocyst stage except in AcH3K18. We speculated that CBHA depresses the expression of certain histone deacetylases, thus limiting the modification of deacetylation and increasing the global acetylation level. The hyperacetylation of histone may affect the chromatin remodeling and provide a more permissible and accessible configuration for transcription factors or chaperones combining (Vignon et al., 2002; Lee et al., 2004; Deshmukh et al., 2012; Mason et al., 2012). Embryonic gene activation (EGA) in pigs happened at the 4-cell stage (Maalouf et al., 2009), and the increase of global histone acetylation was mainly discovered at the 1-cell and 2-cell stages, which suggested that a more “open” chromatin structure might be beneficial for EGA.

To further explore the effect of CBHA on SCNT embryo development, we tested the expression of five important genes at the transcription level in the blastocysts of SCNT embryos, CBHA-SCNT embryos, and IVF embryos. This included two development-related genes: *cdx2* and *pou5f1*, two imprinted genes: *igf2* and *igf2r*, and histone deacetylase 2 (*hdac2*). In IVF embryos, the expression of *cdx2* and *pou5f1* are significantly higher than the control SCNT and CBHA treated SCNT embryos. However, CBHA treatment improved the expression of the two genes to a large extent compared with the control SCNT embryos. *Cdx2* plays a key role in embryo placental development, and in mouse early embryo development, interaction between *oct3*/4 and *cdx2* determines the level of trophectoderm differentiation (Tomanck et al., 1989; Chawengsaksophac et al., 2004; Strumpf et al., 2005). The extremely low expression of *cdx2* in the control SCNT embryos may explain the abnormal placental development in some cloned embryos. While *pou5f1* (*oct4*) is a well-known gene in embryo development, it interacts with regulatory enzymes and epigenetic histone markers in mice. As a central regulator of pluripotency and cell differentiation, it positively modulates the transcription of other recognized stem cell regulators, and it plays a vital role in inner cell mass (ICM) maintenance and ES cell self-renewal (Niwa et al., 2005; Loh et al., 2006). A previous study has shown that in porcine SCNT embryos, the expression of *oct4* and *oct4*-related genes are aberrant (Pesce et al., 2001). It has been reported that in mice, CBHA could improve the derivation efficiency of ESCs from SCNT embryos (Dai et al., 2010). In this process, improved expression of *pou5f1* may act. In our experiment, although still lower than the IVF embryos, the expression of *pou5f1* in CBHA-SCNT embryos was significantly increased compared to the control SCNT embryos. For the importance of embryo development, the two best studied imprinted genes, *igf2* and *igf2r,* have been discussed here. It has been reported that these two genes are related to EGA and involved in fetal growth regulation and are essential for normal development (Lee et al., 2006). Aberrant expression patterns of these imprinted genes may be responsible for the abnormal development found in fetuses and offspring originating from SCNT embryos (Latham et al., 1994; Yang et al., 2005). After CBHA treatment, we observed that the expression of *igf2* increased to the same level as the IVF embryos, yet the expression of *igf2r* was not affected. It can be concluded that CBHA can improve the transcription activity of these genes, which should be related to the more “open” chromatin configuration. CBHA “corrects” the expression of the pivotal genes. It is known that histone deacetylase (*hdacs*) and histone acetylase (*hats*) mediate histone acetylation-deacetylation (Peserico et al., 2011). Previous studies on IVF and *in vivo* porcine embryos have reported that *hdac2* exhibited a low level of expression at 4-cell stage, which steadily increased to its maximum level at the blastocyst stage, however this trend was reversed in SCNT embryos (Kumar et al., 2007; Peserico et al., 2011). Indeed, our results showed that the highest expression of *hdac2* occurred in the IVF blastocyts. However, we also observed that the expression of *hdac2* was hardly affected by the CBHA treatment. This may be because the *hdac2* was not the target protein of CBHA and it is not the only deacetylase to regulate histone deacetylation.

Nevertheless, unlike the improved development *in vitro*, full term development efficiency was hardly affected by the CBHA treatment. The cloning efficiency between CBHA-SCNT and the control SCNT groups is not significantly different. One reason may be that the sample volume was not sufficient to show the difference between the two groups. Another may be because we transferred the embryos at the 2-cell stage. While the *in vivo* and *in vitro* conditions for pre-implanted embryo development are different, the *in vivo* environment may correct the aberrant development from the 2-cell stage onwards to some extent. Another possibility is that the donor cells we transferred are in a good state, therefore the embryos are well developed *in vivo* even without the CBHA treatment. Thus in our experiment, the piglet birth rate is much higher than other reports (0–3.7%) (Zhao et al., 2010), regardless of the CBHA-SCNT (4.2%) or the control SCNT groups (4.4%).

To sum up, our present study indicates that, as a HDACi, CBHA can improve the *in vitro* development of pig nuclear transfer embryos. The presence of CBHA can increase the global acetylation level in AcH3K9, AcH3K18, and AcH4K16. It influences the modification of chromatin structure and improves the expression of important genes in development and imprinting, rendering a more similar expression profile to IVF ones. It enhances the nuclear reprogramming and *in vitro* development of pig SCNT embryos, which may provide better qualified embryos for ES cell derivation in the case of low quality donors. Further studies are needed to discuss the effect of CBHA on chromatin configuration modifications.

## Materials and methods

### Chemicals

All chemicals were purchased from Sigma Chemical Co. (St. Louis, MO), unless otherwise stated.

### Preparation of somatic cells for SCNT

Porcine fetal fibroblast cells were established from a 35-day old fetus. Cells at passage 2–5 were grown to 90% confluency in Dulbecco’s Modified Eagle Medium (DMEM; Gibco BRL, Life Technologies, Grand Island, USA) and supplemented with 10% fetal bovine serum (Gibco BRL, Paisley, UK). Fibroblast cells were dissociated with 0.25% trypsin-EDTA (Invitrogen, Carlsbad, CA, USA) at 37°C for about 3–5 min, then transferred to a centrifuge tube with Hepes-buffered Tissue Culture Medium 199 (TCM-199; BioWhittake, Walkersville, MD, USA) and supplemented with 2% cattle serum (CS; Danish Veterinary Institute, DTU, Frederiksberg, Denmark). The suspension was stored in ice until use.

### *In vitro* maturation of oocytes

Ovaries were collected from prepubertal gilts at a local slaughterhouse and transported to the laboratory at 37°C. Follicles between 3 mm and 6 mm in diameter were aspirated with an 18-gauge needle attached to a 10-mL syringe. The cumulus-oocyte complexes (COCs) in the follicular fluid were allowed to settle by gravity. The COCs were rinsed in Hepes-buffered Tyrode’s medium containing 0.01% PVA three times. Only the COCs with multiple layers of intact cumulus cells and uniform ooplasm were selected for *in vitro* maturation (IVM). After washing three times in IVM medium, a group of 50 COCs was placed into each well of twenty-four-well cell culture plates containing 500 μL of IVM medium. The COCs were matured for 42 h at 38.5°C and 5% CO_2_ in air with 100% humidity. Matured COCs were then vortexed in 0.1% hyaluronidase in Hepes-buffered Tyrode’s medium containing 0.01% PVA for 3 min to remove the cumulus cells. Only the matured oocytes having an extruded first polar body (PB) with uniform cytoplasm were used for the SCNT or IVF embryos.

### Somatic cell nuclear transfer

Matured oocytes used for SCNT were placed in manipulation medium (MAN) supplemented with 7.5 μg/mL Cytochalasin B, and overlaid with warm mineral oil. Oocytes were enucleated by aspirating the polar body (PB), metaphase II chromosomes, and a small amount of surrounding cytoplasm using a beveled glass pipette with an inner diameter of 17–20 μm. A single intact donor cell was injected into the perivitelline space and placed adjacent to the recipient cytoplasm. Karyoplast-cytoplast complexes (KCCs) were placed into embryo culture medium until fusion and activation occurred. The fusion and activation of the KCCs were accomplished with two direct current pulses (1 s interval) of 1.2 kV/cm for 30 microseconds provided by Eppendorf Multiporator in fusion medium (0.3 mol/L mannitol, 1.0 mmol/L CaCl_2_, 0.1 mmol/L MgCl_2_, and 0.5 mmol/L Hepes [pH adjusted to 7.0–7.4]). Oocytes were then incubated for 20 min in PZM3 and evaluated for fusion under a stereomicroscope. Only the fused embryos were placed into four-well cell culture plates containing 500 μL of PZM3 at 38.5°C and 5% CO_2_ with 100% humidified air.

### Post-activation treatment and embryo culture

Stock solutions of CBHA were dissolved in dimethyl sulfoxide (DMSO) at 100 mmol/L and stored at −80°C. Following electrical activation, the SCNT embryos were treated with various concentrations (from 0.02 μmol/L to 400 μmol/L) of CBHA for different times (from 16 h to 24 h) in PZM3. After treatment, embryos were washed three times and then transferred into a four-well cell culture plate containing 500 μL PZM3 medium. They were cultured at 38°C and 5% CO_2_ with 100% humidity either overnight or for 6 days. Cleavage and blastocyst formation were evaluated on days 2 and 7, respectively, with the day of SCNT designated day 0.

### Embryo transfer

Day 2 SCNT embryos were transferred to the oviducts of surrogates on the day of, or 1 day after, the onset of estrus. Pregnancy was diagnosed on day 25, and the surrogates were checked regularly at 2-week intervals by ultrasound examination. All of the cloned piglets were delivered by either natural birth or cesarean section on day 117 of gestation and hand raised. All animals were treated according to preapproved institutional animal care and use protocols.

### Blastocyst cell number counting

Expanded day 6 blastocysts derived from SCNT were selected for cell number comparison. After being fixed in 4% paraformaldehyde in PBS for 15 min at room temperature, embryos were mounted on slides in mounting medium containing 4,6-diamidino-2-phenylindole. At least 10 oocytes or embryos were processed for each separate sample, and the experiments were replicated three times. Slides were analyzed under an epifluorescent microscope (LEICA DM2500) equipped with a digital camera.

### Detection of histone acetylation in SCNT embryos

Embryos treated without (control) or with the optimal CBHA concentration were collected at different stages of development: 1-cell stage (4 h after fusion); 2-cell stage (28–40 h after fusion); and blastocyst (140–150 h after fusion); and fixed in 4% paraformaldehyde in phosphate buffered saline (PBS) for 1 h at 4–8°C. Subsequently, embryos were washed in PBS containing 0.1% Tween 20 (TPBS) for 30 min and permeabilized by 1% Triton X-100 in PBS overnight at 4–8°C. After washing, unspecific antibody binding was blocked by incubation in 2% bovine serum albumin (BSA) in PBS for 1 h, followed by 1 h incubation with primary antibody at RT (anti-acH3K9, 1:500; anti-acH4K16, 1:500; anti-acH3K18, 1:500). After washing, the embryos were incubated for 1 h with Alexa Flour 594-conjugated secondary antibody (1:100, Jackson, USA). Immunofluorescent labeling of AcH3K9, AcH3K18, and AcH4K16 in TSA treated and control embryos were evaluated under Zeiss LSM 510 Laser Scanning Microscope System with the same exposure times and adjustments of the microscope. Fluorescence was measured by analyzing the embryo pictures with Image J software. Average pixel intensity value was measured in 5 random nucleoplasm regions (excluding the nucleolar regions) subtracting the average value measured in the same process within the cytoplasm. Negative control was performed by omitting the primary antibody and led in all cases to the lack of labeling.

### Quantitative PCR

At day 15, seven blastocysts were used per sample. The total RNA of the embryos was isolated using the Phenol-chloroform extraction protocol. The mRNA levels were quantified using SYBR Green Jump Start Taq Ready Mix on a real-time PCR detection system at the following thermal cycling conditions: 94°C for 2 min, followed by 40 PCR cycles of 94°C for 15 s, 60°C for 30 s, and 72°C for 30 s. Transcripts were quantified in 3 replicates for each sample and calculated relative to the transcription of the housekeeping gene, β-actin, in every sample. The 2^−△△CT^ was used to quantify the relative mRNA levels. The primers used in the quantitative PCR are listed in the Table S1.

### Statistical analysis

Data were analyzed using SAS (9.1) with one-way ANOVA. A probability of *P* < 0.05 was considered to be statistically significant.

## Electronic supplementary material

Below is the link to the electronic supplementary material.

Supplementary material 1 (PDF 14 kb)

## Abbreviations

CBHA: *m*-carboxycinnamic acid bishydroxamide
COCs: cumulus-oocyte complexes
EGA: embryonic gene activation
ESCs: embryonic stem cells
HDACi: histone deacetylase inhibitors
iPSC: induced pluripotent stem cells
KCCs: karyoplast-cytoplast complexes
PBS: phosphate buffered saline
SCNT: somatic cell nuclear transfer
