# Supplementary material for: Epigenetic reprogramming, gene expression and in vitro development of porcine SCNT embryos are significantly improved by a histone deacetylase inhibitor—m-carboxycinnamic acid bishydroxamide (CBHA)
Source: Protein Cell. 2014 Mar 14;5(5):382–93. doi: 10.1007/s13238-014-0034-3 (PMC3996156; doi:10.1007/s13238-014-0034-3)
Supplement: Supplementary file 1 — Supplementary material 1 (PDF 14 kb) [file 13238_2014_34_MOESM1_ESM.pdf]

Table S1. Primer sequence for RT-PCR and Q-PCR

Gene name	Forward primers	Reverse primers
**CDX2**	CGGAACCTGTGCGAGTGGA	TGCCAGCTCGGCCTTTCT
**HDAC2**	ACAGGAGACTTGAGGGAT	CACATTTAGCGTGACCTT
**IGF2**	GTGCTGCTATGCTGCTTACCG	CCGCAGACAAACTGGAGGG
**IGF2R**	CGGAGTTCAGCCACGAGA	CACCAGCAGCAGACTAAGGAT
**POU5F1**	GTTCAGCCAAACGACCATCT	CTCTGCCTTGCATATCTCCTG
**β-actin**	TGCTGTCCCTGTACGCCTCTG	ATGTCCCGCACGATCTCCC
